# The Nature of Shock and Allied Conditions

**Published:** 1893-06

**Authors:** 


### The Nature of Shock and Allied Conditions.

A study of the subject has led to the following conclusions:

1. Shock is not due to a spasm of the heart or vessels.

2. It is often due to a paresis of the vagus nerve, caused either (1) *directly* by emotions, severe jars, etc., or (2) by *reflex* influence from injuries of other nerves (certain poisons by *direct* action also cause symptoms much like those of shock.)

3. It is questionable if shock is ever due to the inhibitory action of the vagus on the heart's action, but possibly some cases of sudden death from shock may be explained in this way.

4. In many cases shock is due to the dilatation of the vessels in the abdominal cavity, which is often accompanied by a paresis of the vagus nerve.—*Phila. Medical News*, Dec. 3d, 1892.

The following is taken from a paper on the necessity for prevention against cholera, by James F. Hibberd, M.D.

Bacteria are immense numbers of microscopic vegetables so small that the highest magnifying power has found specimens too minute for correct description. Medical bacteriologists for convenience distribute these microscopic plants into three classes—a round one is a micrococcus, a straight filament is a bacillus, and a spiral filament is a spirillum. These designations signify nothing but form and are simple aids to reference, but other attributes are many and important to sanitarians. Bacteria are found everywhere; in the water, in the atmosphere, in the soil, on and in man and all animals, and on everything man and animals eat. Most of them are harmless, many of them are salutary and contribute to animal health and longevity, including man, and only fifteen of them have so far been satisfactorily proven to be the direct cause of disease in man, each of which excites a specific disorder that has no other origin. It is on these lines that physicians and sanitarians investigate and consider bacteria, but botanists make extensive subdivisions of them into genera, species and varieties according to the rules of botanical science.

This microbe is, however, a delicate plant, and can propagate only within relatively narrow limits. It can not survive in a temperature above 126° F., nor below 57°, and its active life is confined to a temperature between 70° and 108° F. It cannot multiply, though it can live, in pure water, but in water containing organic matter in solution it flourishes in a high degree, as it does also in damp soil containing organic matter. Hence foul lakes, ponds, rivers, wells and cisterns, and wet soils, drains and gutters, and sewage are favorite habitats and breeding places for the cholera spirillum. As already stated, it cannot live in a temperature below 57° nor above 126° F. Drying kills it, the most contaminated clothing, bedding or other fabric, or soil, when completely dried, are clear of every living cholera germ, nor can it survive in acids or strong alkalies. It is, in fact, a very tender and fastidious plant, must have temperature and food just suited to its taste, or it will perish. Surely an enemy so dreaded as the cholera spirillum, whose frailty we clearly understand, and whose many vulnerable points we well know, ought to fall an easy victim to our cultured and energetic germicidal skill.

Some criticism having been made on the above remarks a letter was addressed to Dr. Hibberd, by Dr. D. N. Berg to which the following explanatory reply was given:

RICHMOND, IND., MAY 5, 1893.

D. N. Berg, Indianapolis, Ind.:

*Dear Sir:*—Your note of the 3d inst. states that parties listening to my address before the sanitary conference on the 27th ult. have since made a double inquiry, which may be restated thus: If my figures of the temperature limits of the life and activity of the cholera spirillum are correct how is it that cholera has prevailed to some extent in Russia all winter? I reply, there is a slight but immaterial difference in the observations of bacteriologists touching the extremes of temperature within which the cholera germ may live and multiply. Sternberg in his great work, "Manual of Bacteriology," published within the year, says, page 504: "The thermal death point of the cholera spirillum in recent cultures in flesh-peptone-gelatine as determined by the writer (1887) is 52 degrees c." The equivalent of 52 degrees centigrade is 125.6° Fahrenheit. On page 501 he says the germ "does not grow at a temperature above 42 degrees or below 14 degrees c," equivalent to 107.6 degrees and 57.2 degrees Fahrenheit respectively, and on the same page he describes the "gelatine plate culture at 22 c," equivalent to 71.6° Fahrenheit. Omitting fractions these are the figures used in my communication to the sanitary conference.—Yours respectfully,

JAMES F. HIBBERD.

